# The Effect of Non-Surgical Periodontal Treatment on Patients with Combined Refractory Arterial Hypertension and Stage III, Grade B Periodontitis: A Preliminary Prospective Clinical Study

**DOI:** 10.3390/jcm12134277

**Published:** 2023-06-26

**Authors:** João Victor Soares Rodrigues, Marina Módulo Cláudio, João Paulo Soares Franciscon, Rossana Abud Cabrera Rosa, Thamiris Cirelli, Rafael Scaf de Molon, Carlos Marcelo S. Figueredo, Valdir Gouveia Garcia, Leticia Helena Theodoro

**Affiliations:** 1 Department of Diagnostic and Surgery, School of Dentistry, São Paulo State University (UNESP), Araçatuba 16015-050, SP, Brazil; joao.vic.t@hotmail.com (J.V.S.R.); marinamodoloc@gmail.com (M.M.C.); joao.franciscon@unesp.br (J.P.S.F.); rossana@unisalesiano.com.br (R.A.C.R.); rafael.molon@unesp.br (R.S.d.M.); leticia.theodoro@unesp.br (L.H.T.); 2 Center for Dental Assistance to Persons with Disabilities (CAOE), School of Dentistry, Araçatuba 16015-050, SP, Brazil; 3 Department of Dentistry, University Center of Associated School—UNIFAE, São João da Boa Vista 13870-377, SP, Brazil; thamiriscirelli@gmail.com; 4 School of Medicine and Dentistry, Griffith University, Brisbane, QLD 4111, Australia; 5 Division of Oral Diseases, Department of Dental Medicine, Karolinska Institutet, OF Odontologi, OF Orala sjukdomar, 171 77 Stockholm, Sweden; 6 Latin American Institute of Dental Research and Education (ILAPEO), Curitiba 80710-150, PR, Brazil; vg.garcia@uol.com.br

**Keywords:** periodontitis, periodontal disease, hypertension, dental scaling, clinical trial

## Abstract

We sought to evaluate the effects of non-surgical periodontal treatment (NSPT) on periodontal clinical parameters, systemic blood pressure (BP), and plasma levels of systemic inflammation markers in patients with combined refractory arterial hypertension (RAH) and stage III grade B periodontitis. Twenty-seven participants with RAH and periodontitis received NSPT. The analyzed clinical parameters were probing depth (PD), clinical attachment level (CAL), bleeding on probing (BOP), and plaque index (PI). An assessment was performed for systemic BP, complete blood count, coagulogram, creatinine measurement, C-reactive protein (CRP), glycated hemoglobin, cholesterol, glutamic oxaloacetic transaminase, glutamate pyruvic transaminase, waist–hip ratio, and body mass index. In the follow-up period, twenty-two patients were evaluated at baseline and after 90 and 180 days. The data were submitted for statistical analysis (α = 0.05%). As expected, the clinical results showed a significant improvement in the percentages of PI, BOP, PD, and CAL, which were statistically significant at 90 and 180 days (*p* < 0.0001). Importantly, NSPT significantly reduced the blood level of CRP (*p* < 0.02). However, no significant reduction in BP parameters was noted in the evaluated follow-up periods. NSPT, despite the benefits in periodontal clinical parameters, reduced the plasma level of CRP but not the BP in patients with combined RAH and periodontitis.

## 1. Introduction

Arterial hypertension (AH), a complex multifactorial clinical condition, is characterized by an elevation of blood pressure at levels ≥ 140 and 90 mmHg [1]. It is estimated that 31.1% of the global population is diagnosed with AH [2]. Cardiovascular diseases (CVD) have persisted as the leading causes of death worldwide and significantly contribute to loss of health and excess health system costs [3]. High systolic blood pressure (SBP) rests as the foremost changeable risk factor globally for attributable cardiovascular deaths, accounting for 11.3 million deaths overall in 2021 [4]. To date, the mechanisms that can fully explain the increase in blood pressure (BP) is not totally elucidated [5]. Refractory arterial hypertension (RAH) is defined as an increase in BP in patients using three or more antihypertensive medications, including diuretics [6].

Periodontitis is a multifactorial chronic inflammatory disease associated with a dysbiotic biofilm and characterized by the progressive destruction of supporting periodontal tissues [7]. Dysbiosis results from inflammation-mediated microbial succession in periodontitis, in which disease-related species appear over time in the periodontal pocket [8]. Periodontitis is one of the most common chronic inflammatory conditions with a prevalence rate of around 50% [9], in which the prevalence of its severe form account for 11% of the global population, affecting approximately 734 million people worldwide [10]. Periodontitis is considered the sixth most common epidemic disease globally [11], and the leading cause of teeth loss in the adult population [12]. Moreover, periodontitis has been considered a risk factor for other chronic inflammatory diseases, such as cardiovascular inflammation and endothelial dysfunction [13,14], rheumatoid arthritis [15,16], and diabetes mellitus [17,18]. Therefore, if a causal relationship could be shown, periodontitis would be associated with an increasing overall burden of hypertension and other non-communicable chronic diseases, and oral health assessment and management of oral inflammation could have an essential role in preventing hypertension and its complications [11]. CVD has recently been associated with periodontitis in a joint focused workshop of the European Federation of Periodontology and the World Heart Federation (WHF) in 2019 highlighting the positive association between the two conditions and the overall burden of hypertension and other non-communicable conditions, such as periodontitis [19]. Moreover, periodontitis was independently associated with CVD and other conditions, and therefore the implementation of strategies for early detection of periodontitis in primary care centers plays an important role in preventing such conditions in dental settings [20]. Despite the current understanding regarding the effect of periodontitis on RAH, the nature of this association is still poorly understood.

The underlying mechanism that links periodontitis and CVD is the increase in low-grade inflammation caused by periodontitis, which enhances the systemic body inflammation, acting as a silent killer [21]. Moreover, the systemic spreading of periodontal pathogens, especially *P. gingivalis,* from the ulcerated epithelium through the bloodstream has been found in circulating leukocytes and was also found in atherosclerotic plaques acting as a pro-atherogenic stimuli [21,22]. Furthermore, might also alter the gut microbiota, thereby leading to epithelial permeability and endotoxemia [21,22].

Several cohort studies have associated periodontitis with hypertension [23,24], while others have pointed to the relationship between periodontitis and the risk of AH [25,26,27]. In a recent systematic review, Munoz Aguilera et al. (2020) suggested a linear association between periodontitis and the risk of AH showing that periodontitis diagnosis increased the likelihood of AH occurrence (OR = 1.68) [28]. Patients with periodontitis exhibited a higher mean of SBP and diastolic blood pressure (DBP) when compared with non-periodontitis patients in prospective studies [27]. Of importance, 5 out of 12 interventional studies confirmed a reduction in blood pressure levels following periodontal therapy, ranging from 3 to 12.5 mmHg of SBP and from 0 to 10 mmHg of DBP [28]. These findings parallel observations made by previous studies showing that patients subjected to non-surgical periodontal treatment (NSPT) showed improved periodontal clinical parameters and reduced plasma levels of inflammatory markers [29,30,31,32].

Vidal et al. (2011) found that periodontitis is a risk indicator for hypertensive patients, and there are indications that systemic inflammation is associated with both conditions [33]. A recent study tested the association of periodontal inflammation and bleeding on probing (BOP) using the NHANES III database [34]. Compared to no inflammation, severe periodontal inflammation and gingival bleeding were associated with 43% and 32% higher odds of high/uncontrolled BP (≥130/80 mmHg), and with higher SBP by approximately 4 and 5 mmHg, respectively [34]. They concluded that local inflammation might contribute to the inflammatory burden to mediate this association [34]. 

Since there is still limited evidence suggesting that successful NSPT can improve systemic BP post-treatment in RAH patients [29,30,31], we hypothesize that NSPT in RAH patients would enhance the control of BP and a reduction in the plasma levels of systemic inflammatory markers. Therefore, the objective of this study was to clinically evaluate the effect of NSPT on clinical periodontal parameters, including BOP, clinical attachment level (CAL), probing depth (PD) and plaque index (PI), systemic BP (systolic and diastolic) and the plasma levels of systemic inflammation markers (protein C-reactive) in patients with RAH and stage III, grade B periodontitis.

## 2. Materials and Methods

### 2.1. Study Population

A prospective, interventional cohort clinical study was developed and performed in a single center with follow-ups of 90 and 180 days. The study was carried out between August 2019 and May 2021. This study was approved by the Human Research Ethics Committee of the Dentistry School of Araçatuba (CAAE 14338819.5.0000.5420) and was registered with the Brazilian Registry of Clinical Trials (Registration Number: RBR-9d78qy). The report follows the rules of the TREND Statement. 

### 2.2. Participants

To be included in this study, participants had to meet the following inclusion criteria: a diagnosis of stages III and/or IV, grade B periodontitis [7] and be diagnosed with RAH ≥140 mmHg and 90 mmHg for five years, using three or more antihypertensive medications [6]. Briefly, stage III of periodontitis is associated with severe periodontitis, i.e., CAL ≥ 5 mm and PD ≥ 6 mm, and the disease grade corresponds to the disease progression rate over the last 5 years, according to the World Workshop on the Classification of Periodontal and Peri-Implant Diseases and Condition in 2018 [7].

Exclusion criteria were defined as smokers or former smokers; individuals with anemia; active cancer; blood disorders; pregnancy; chronic kidney disease; type II diabetes mellitus (HbA1c > 7.0); bacterial endocarditis [30]; having received some type of periodontal therapy in the last 6 months; current use of medication such as antibiotics or anti-inflammatories; systemic conditions that affect periodontal progression or treatment [30]; individuals undergoing chemotherapy or head and neck radiotherapy; medical disorders that required antibiotic prophylaxis or that could influence treatment response; as well as alcoholism and illicit drug use.

**Figure 1:**
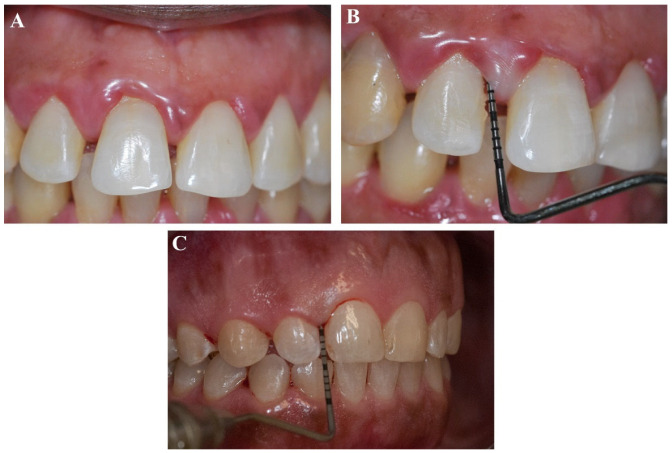
Clinical examination methods: (**A**) plaque index (PI), (**B**) probing depth (PD), (**C**) clinical attachment level (CAL), and bleeding on probing (BOP).

The participants were recruited from patients at the Periodontics Clinic of the Araçatuba School of Dentistry-UNESP. First, they completed a full questionnaire about their general and oral health to assess whether they met the inclusion criteria. If positive, a periodontal assessment (PI, BOP, PD, and CAL) for all the included patients was accompanied (Figure 1). The full questionnaire the patients filled out during the visits to the dental office included socio-demographic characteristics, systemic conditions, and use of medications.

### 2.3. Primary and Secondary Clinical Outcomes

The primary outcomes evaluated in this study were the percentage of sites with CAL gains and a reduction in the number of sites with PD ≥ 5 mm, the clinical parameters of PD, BOP, and PI. The secondary outcomes evaluated in this study were BP and systemic inflammatory markers. 

The clinical examination of all participants was performed by a single calibrated examiner (JVSR) at baseline and 90 and 180 days post-treatment. In the clinical examination, the visible PI (present or absent) was registered at the six sites of all teeth in the oral cavity. PD (the distance between the gingival margin to the bottom of the periodontal pocket), BOP (bleeding during periodontal probe—present or absent), and CAL (the distance between the cement–enamel junction to the bottom of the periodontal pocket) examinations were also performed at all six sites of each tooth in the oral cavity. These clinical parameters were obtained using a millimeter periodontal probe (PCPUNC-15, Hu-Friedy, Chicago, IL, USA).

### 2.4. Blood Pressure Analysis

In addition to the previous medical diagnosis of RAH, the BP of each participant was obtained through measurement with a non-invasive digital electronic sphygmomanometer (Digital Automatic Arm Blood Pressure Monitor—Omron), validated and calibrated. The BP measurement was performed by explaining the procedure and keeping the participants at rest for three to five minutes in a calm environment before verification. They were instructed not to talk during the measurement and not to have a full bladder. The examiner also asked about any physical exercise practiced less than sixty minutes before the measurement and ingestion of alcohol, coffee, or food less than thirty minutes before BP measurement. The measurement was performed with the participant seated, legs uncrossed and feet flat on the floor, back leaning, and with the arm positioned at heart level and supported with the palm facing up, clothes not constricting the upper arms. Measurements were taken on both arms, twice with a one-minute interval between each measurement, after which the highest value was used [1]. In addition, information was obtained about medications and dosages to control AH or other associated systemic diseases.

### 2.5. Bodyweight Analysis

Participants underwent analysis of the waist–hip relationship (WHR = waist [cm] ÷ hip [cm]) using a tape measure, considering limit values for men and women at 0.85 and 0.90, respectively [35]. Furthermore, body mass index (BMI) values were obtained using anthropometric scale (digital anthropometric scale 200 kg/100 g, Welmy), through the values of weight (kg) and height (m), using the calculation BMI = weight [Kg]/height^2^ [m^2^] [36].

### 2.6. Blood Analysis

A specialized clinical analysis laboratory collected peripheral blood samples 7 days before the clinical evaluations. The requested tests were complete blood count, coagulogram, CRP, fasting and estimated blood glucose, glycated hemoglobin (HbA1c), total cholesterol, oxalacetic glutamic transaminase (OGT), pyruvic glutamic transaminase (PGT) and creatinine. The determination of positive CRP was characterized by levels equal to or above 6 mg/l, with indices below that threshold considered negative [37]. The above exams were requested at baseline and the 90 and 180 days follow-ups.

### 2.7. Experimental Design and Treatment Protocol

Fifteen days before treatment, the clinical periodontal examinations were performed (baseline) using a millimeter periodontal probe (PCPUNC-15, Hu-Friedy, Chicago, IL, USA). Immediately after this procedure, participants received detailed information about the etiology of periodontal disease and oral hygiene instructions (OHI), including toothbrushes, dental floss, and interproximal brushes as needed. This instruction was repeated at each visit.

The initial treatment of periodontitis consists of the NSPT of subgingival instrumentation (SI) in combination with OHI [38]. SI refers to the subgingival scaling and root planing (below the gingival margin). The participants underwent an SI session of 1.5 h, using Gracey and McCall manual curettes (Hu-Friedy Chicago, IL, USA), in line with the recommendations of the Guideline for Clinical Practice [38]. The SI procedures were performed by a single and experienced periodontist (JVSR) that possesses a specialist and MS degree in periodontics and is currently enrolled in a PhD program. All the SI procedures were performed without local anesthesia.

One week after SI, all participants were visually inspected to assess possible undesirable signs and symptoms. Subsequently, follow-ups took place after 90 and 180 days, during which clinical exams were performed and laboratory tests requested, following the same parameters as in the initial exams. In these reassessments, supragingival plaque control and OHI were given according to each participant’s needs [30,32].

### 2.8. Statistical Analysis

#### Sample Calculation

Based on previous studies [30,32], sample size was calculated (Biostat 5.0, Mamirauá Institute, AM, Brazil) with a test power of 80% and the significance level set at 5%, to recognize a significant difference of 1 mm in PD. Considering a possible loss of 20%, the inclusion of 27 participants was recommended.

The demographic data, periodontal clinical parameters, complementary exams obtained from blood tests, physical exams, and BP were submitted to descriptive and analytical statistical analysis using software package (GraphPad Prism versão 8.3.4, San Diego, CA, USA). The data showed a normal distribution after the normality test (Shapiro–Wilk). Periodontal clinical variables, blood tests, and physical and BP data were compared between periods (baseline, 90 and 180 days) and presented as means and standard deviation (SD). The data were then submitted to the ANOVA test, followed by Tukey’s post hoc test. The categorical variable CRP was compared using the chi-square test. All analyses were performed at a significance level of 5%.

A linear regression analysis was performed with the help of STATA 12.0 for Windows (Statistics/Data Analysis, Stata Corporation, College Station, TX, USA) to evaluate the effects of the different periods on the periodontal clinical parameters, biochemical and physical exams, and BP parameters. Logistic regression analysis was then performed to evaluate the effect of different periods on periodontal clinical parameters (BOP, CAL ≥ 6 mm) and SBP and DBP. The results were binary (presence or not): (i) severity of the periodontal disease, with a predominance of deep pockets (CAL ≥ 6 mm or BOP ≥ 30% of the evaluated sites) [39]; or (ii) changes in BP levels (SBP ≥ 140 mmHg or DBP ≥ 90 mmHg) [1].

## 3. Results

### 3.1. Examiner Calibration

In the pre-experimental period, the examiner’s calibration procedures were performed through a clinical periodontal evaluation of six participants. PD and CAL parameters were obtained on two separate occasions (7-day intervals). Data were tabulated and submitted to the Kappa agreement test [40]. The obtained value of 0.88 is representative of a significant intra-examiner agreement.

### 3.2. Participants Sample

**Figure 2:**
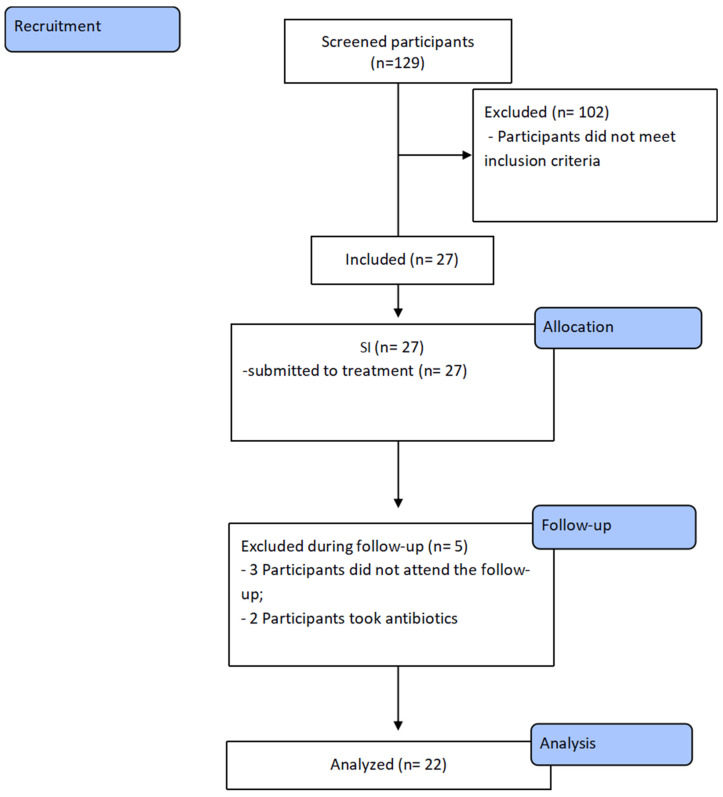
Flowchart of the study.

Following the initial screening, 129 participants were recruited (Figure 2). Of these, 102 were excluded because did not meet the inclusion criteria. The remaining 27 patients were enrolled in the study presenting with RAH and stages III and IV grade B periodontitis.

Thirteen men and fourteen women aged 45 to 71 years underwent NSPT and were individually informed about the nature of the study and signed an informed consent form. During the follow-up visit, five participants were excluded due to antibiotic use (2) and non-attendance at follow-ups (3). Thus, the final assessment in 90 and 180 days consisted of 22 participants (Figure 2).

Table 1 presents the demographic characteristics of the 22 participants who make up the study’s final sample, 10 men (45.45%) and 12 women (54.55%) with a mean age of 59.9 years. No statistically significant difference was observed in the distribution between men and women.

The antihypertensive drugs most used by the participants were losartan (63.3%) and atenolol (15.40%). Regarding diuretics, 45.45% of the participants used hydrochlorothiazide, and 22.72% used furosemide. Regarding anticoagulants, 18.18% used the drug acetyl salicylic acid. To reduce blood cholesterol levels, 45.45% of the participants used simvastatin, and for glycemic control, 18.18% used metformin.

### 3.3. Clinical Parameters 

Regarding periodontal clinical parameters, there was no significant difference in the number of teeth between the evaluated periods. In the analysis of %PI, BOP, PD ≤ 4 mm, PD ≥ 5 mm, and CAL ≤ 3 mm, there was a statistically significant reduction at 90 and 180 days compared to baseline. Comparing the percentage of sites with a CAL 4–5 mm (*p* = 0.11) and CAL ≥ 6 mm (*p* = 0.59), no significant difference between the evaluated periods was found (Table 2).

Table 3 presents the linear regression analysis of individuals in the different periods analyzed after NSPT (90 and 180 days) to verify its effect on the study variables. Similar to what is presented in Table 2, the main significant results were found in the periodontal clinical parameters, i.e., PI, BOP, PD ≥ 5 mm, and CAL ≤ 3 mm presenting an inversely proportional decrease as the follow-up time increased. However, there was an increase in the percentage of sites with PD ≤ 4 mm, per evaluation period, confirming the improvement of deep periodontal pockets after NSPT.

Table 4 presents the logistic regression analysis between the different periods. To categorize periodontitis according to the severity of gingival tissue inflammation, the subjects were divided according to the presence of BOP in ≥30% of the evaluated sites or the severity with a presence of CAL ≥ 6 mm. It was observed that for both the baseline versus 90 days and baseline versus 180 days comparisons, individuals were less likely to present BOP in ≥30% of sites (OR 0.02, 95% CI 0.002- 0.29, *p* = 0.001; OR 0.05, 95%CI 0.009–0.26, *p* = 0.000, respectively). Regarding CAL ≥ 6 mm, no significant differences were found at 90 days (OR 0.54, CI 95%: 0.18–1.68, *p* = 0.28), or 180 days (OR 0.61, CI 95%: 0.19–1.94, *p* = 0.41).

### 3.4. Blood Pressure 

In the analysis of systemic BP parameters, no statistically significant differences were observed in any variable (*p* > 0.05). No significant reductions were observed at 90 and 180 days, when compared to baseline, in the data of SBP (*p* = 0.28), DBP (*p* = 0.39), mean arterial pressure (MBP; *p* = 0.08), and differential pressure (*p* = 0.09; Table 2). Statistically significant differences were not observed in systemic BP parameters in the different evaluated periods in the linear regression analysis (Table 3).

To assess systemic BP parameters, the participants were divided according to the changes found, SBP ≥ 140 mmHg or DBP ≥ 90 mmHg, but no statistically significant differences were found in SBP at 90 days (OR 0.48, 95% CI: 0.15–1.61, *p* = 0.24) and 180 days (OR 0.83, 95% CI: 0.26–2.65, *p* = 0.75), or in the DBP at 90 days (OR 1.17, CI 95%: 0.34–1.02, *p* = 0.79) and 180 days (OR 1.97, CI 95%: 0.59–6.63, *p* = 0.27; Table 4).

### 3.5. Blood Analysis

All blood count and coagulogram parameters were within normal ranges. The test results for HbA1c, fasting blood glucose, estimated blood glucose, total cholesterol, creatinine, OGT, and PGT showed no statistically significant reduction in any follow-up period compared to the baseline (Table 2). In the categorical comparison between the periods, it was observed that after NSPT, there was a significant reduction in the inflammatory marker CRP (*p* = 0.02; Table 2). Statistically significant differences were not observed in the blood biomarker exams in the different evaluated periods in the linear regression analysis (Table 3).

### 3.6. Bodyweight 

The outcomes of physical examinations remained constant in all periods, without any significant differences in the data for waist–hip ratio (*p* = 0.59) and BMI (*p* = 0.98) between the evaluation periods (Table 2). Similar to what is presented in Table 2, statistically significant differences were not observed in physical exams in the different evaluated periods in the linear regression analysis (Table 3). This section may be divided by subheadings. It should provide a concise and precise description of the experimental results, their interpretation, as well as the experimental conclusions that can be drawn.

## 4. Discussion

The results of this prospective clinical study showed that NSPT reduced the systemic levels of CRP but did not result in any positive effect regarding the BP levels in patients with combined periodontitis and RAH. Similar to periodontitis, diabetes, and hypertension share common risk factors (aging, smoking, and unfavorable socioeconomic status). Thus, residual confounders can affect the degree of these associations. Importantly, this connection may also be driven by an association between BP changes and other undetected sources or chronic infections [28]. Periodontitis and hypertension are related to chronic immune dysfunction brought on by periodontal inflammation [41].

Good periodontal health may be associated with better BP control during antihypertensive treatment [42]. The effects of NSPT in hypertensive individuals have been reported in a few studies [30,31,32,43]. In our study, the NSPT of individuals with RAH proved to be beneficial during the 90- and 180-day evaluations. SI and OHI led to a significant reduction of PI, PD, BOP, and deep pockets after 90 and 180 days. These residual pockets are an essential indicator of periodontitis persistence and a heightened risk of periodontal disease progression, which requires a repetition of NSPT or surgical treatment [44]. In the present study, a CAL gain was also observed in sites with CAL ≤ 3 mm in the 90 and 180 days periods, compared to the baseline. Such findings confirm that NSPT promotes significant improvements in periodontal clinical parameters [45]. 

In the logistic regression analysis, aimed at the categorization of periodontal disease according to its severity, it can be observed that at both assessment points, patients had lower chances of ≥30% sites with BOP (OR 0.02, 95%CI 0.002–0.29, *p* = 0.00). These finds indicate that NSPT decreases the chances of generalized gingival inflammation in all experimental periods.

The identification of periodontitis as a possible risk factor for hypertension can be explained by some mechanisms [28]. Periodontitis is related to systemic inflammation, and its mediators include CRP, interleukin 6 (IL-6), and tumor necrosis factor alpha (TNF-α), which can affect endothelial function. Clinical evidence has shown that periodontitis affects the whole-body endothelial function, which may, in turn, affect hypertension [13,14]. An experimental study in animals has demonstrated that the immune response to the common periodontal pathogen *Porphyromonas gingivalis* can cause elevated blood pressure, vascular inflammation, and endothelial dysfunction [46]. 

CRP is elevated in hypertensive patients, being one of the determinants of systemic inflammation caused by AH [47]. Furthermore, periodontitis increases the levels of local and systemic inflammatory markers, in addition to causing changes in the neutrophil function [48]. The latter disrupts the regulatory balance by reducing nitric oxide and induces increased IL-6, TNF-α, and blood CRP levels [48]. This generates vascular changes and endothelial cell dysfunction [48]. Interestingly, the categorical variable CRP showed a statistically significant reduction in individuals with RAH at 90 and 180 days after NSPT. However, other studies have not observed a significant reduction in the plasma levels of inflammatory markers [49] in patients with periodontitis or healthy individuals [50]. Therefore, NSPT, leading to reduced clinical and systemic infection and inflammation, seems to improve endothelial function in patients with RAH.

The effects of periodontal treatment on the plasma levels of inflammatory markers are described in the literature. Some studies have shown that plasma and/or serum levels of IL-6, fibrinogen, CRP, other proteins, and interleukins decrease after NSPT in individuals with CVDs [51], coronary artery disease [52], in normotensive and hypertensive individuals [53], and in individuals with RAH [30,32]. The evaluation periods varied between 1 and 6 months after periodontal treatment in the studies mentioned above.

Regarding the biochemical tests for total cholesterol, creatinine, OGT, and PGT after NSPT, it was observed that NSPT did not result in any beneficial effect. Furthermore, NSPT did not influence the levels of HbA1c, estimated glycemia, and fasting glycemia in individuals with RAH. Some studies have demonstrated an improvement in the control of HbA1c levels in diabetic patients following NSPT [54,55]. Therefore, these results demonstrated that other factors should be considered, such as the use of medicines to control blood glucose, dietary habits, and physical exercise.

Our study did not show statistically significant reductions in the physical examinations of WHR and BMI after NSPT in individuals with RAH. The literature has indicated that obesity plays a role in developing AH and CVDs [56]. Some studies found an association between BMI and AH, which indicates that obese patients are at a higher risk of developing AH [57]. A significant association between SBP and BMI has also been observed in hypertensive individuals [36]. This higher rate of obesity may be related to one of the risk factors for the development of non-communicable diseases such as AH and type 2 diabetes mellitus [19]. In addition, it can be observed that participants of this study with RAH were obese and, presented a high cardiac risk factor.

Subgingival periodontal bacteria levels were directly correlated with SBP and DBP and the prevalence of hypertension [58]. Another study demonstrated that systemic exposure to periodontal bacteria is related to hypertension [42]. Studies that evaluated the effect of NSPT on systemic BP are controversial [26,29,30,31,32,52,53,59]. Some studies have demonstrated a BP improvement after NSPT [29,30,31], whereas other studies did not show such improvements [32,52,53,59]. In the present study, in the analysis of systemic BP variables, no significant reductions were observed after NSPT at 90 and 180 days compared to baseline. 

Recent meta-analyses have shown that regular aerobic and aquatic exercises can significantly reduce SBP [60]. Pietropaoli et al. (2018) demonstrated in a clinical study that a combination of lifestyle adjustments and periodontal therapy could help control BP [42]. Given these facts, it can be noted that other individual factors such as antihypertensive drugs, lifestyle, or diet can interfere with systemic BP control in addition to the treatment of chronic inflammatory diseases. Such findings restate the limitation of the inference that periodontal treatment can directly or indirectly influence the control of AH [42].

This prospective interventional clinical study possesses some important caveats. One is the impossibility of controlling variables that can affect the control of systemic AH, such as changes in the use of antihypertensive drugs, diet, or the individual’s emotional state. The present study was carried out during the Covid-19 pandemic, which may have significantly influenced the control of patients’ systemic BP due to the effects of stress, changes in eating and dietary habits, use of medication, a lack of medical control, and a decrease in physical activity [61]. Finally, the lower number of patients included in this study is also a limitation of the experimental design. Therefore, prospective clinical studies with a significant number of participants to perform a global follow-up of AH patients and to observe the benefits of NSPT in reducing the serum levels of inflammatory markers and systemic BP in individuals with RAH and long-term periodontitis are warranted.

## 5. Conclusions

Within the limits of this study, it can be concluded that NSPT effectively improved periodontal clinical parameters and reduced the plasma level of CRP but did not interfere with systemic BP parameters in individuals with combined stage III periodontitis and RAH.

## Figures and Tables

**Table 1:** Demographic characteristics of the studied sample.

Demographic Characteristics	*n* = 22
Age—mean (SD)	59.9 (±8.2)
Sex—*n*, (%)	
Men	10 (45.45%)
Women	12 (54.55%)

Abbreviations: SD = standard deviation.

**Table 2:** Comparison of periodontal clinical variables, results of biochemical tests and blood pressure parameters evaluated in the different evaluation periods.

Evaluated Variables	Baseline *n* = 22	90 Days *n* = 22	180 Days *n* = 22	*p*-Value
Periodontal Clinical Parameters—Mean (SD)				
N° teeth	22.9 (± 4.1)	22.9 (± 4.1)	22.8 (± 4.0)	0.99
PI (% of the sites)	35.7 (±26.2) ^a^	16.0 (±9.6) ^b^	19.8 (±16.5) ^c^	<0.0025
BOP (% of the sites)	0.39 (±0.18) ^a^	0.29 (±0.09) ^b^	0.17 (±0.08) ^c^	<0.0001
PD ≤ 4 mm (% of the sites)	94.1 (±5.74) ^a^	99.1 (±1.42) ^b^	99.13 (±1.61) ^c^	<0.0001
PD ≥ 5 mm (% of the sites)	5.89 (±5.74) ^a^	0.87 (±1.42) ^b^	0.87 (±1.61) ^c^	<0.0001
CAL ≤ 3 mm (% of the sites)	6.05 (±5.37) ^a^	0.89 (±1.45) ^b^	0.96 (±1.75) ^c^	<0.0001
CAL 4–5 mm (% of the sites)	65.1 (±20.14)	74.29 (±18.56)	74.62 (±18.39)	0.1172
CAL ≥ 6 mm (% of the sites)	28.85 (±17.38)	24.81 (±18.59)	24.42 (±17.63)	0.5968
Biochemical and physical exams—Mean (SD)				
HbA1_c_ (%)	5.78 (±0.52)	5.83 (±0.44)	5.73 (±0.52)	0.7296
Fasting glucose (mg/dL)	103.3 (±14.89)	106.0 (±14.20)	107.0 (±13.12)	0.6814
Estimated glucose (mg/dL)	116.9 (±16.34)	114.0 (±13.15)	114.9 (±13.65)	0.8091
Total cholesterol (mg/dL)	178.1 (±40.07)	195.5 (±47.94)	171.2 (±29.41)	0.2478
Creatinine (mg/dL)	0.94 (±0.32)	0.94 (±0.26)	0.97 (±0.35)	0.9222
PGT (U/L)	23.55 (±9.26)	22.66 (±7.75)	2196 (±8.67)	0.8321
OGT (U/L)	25.10 (±8.77)	23.24 (±6.67)	25.27 (±7.36)	0.8929
Waist hip ratio (cm)	0.97 (±0.08)	0.97 (±0.09)	0.95 (±0.06)	0.5930
BMI (Kg/m^2^)	29.41 (±4.61)	29.66 (±4.62)	29.53 (±5.21)	0.9823
C-reactive protein n, (%)				
Positive	5	0	1	0.0213
Negative	17	22	21	
Blood pressure parameters—Mean (DP)				
SBP (mmHg)	139.6 (±15.24)	132.9 (±15.46)	136.2 (±10.61)	0.2878
DBP (mmHg)	79.50 (±12.35)	77.45 (±17.65)	83.14 (±9.54)	0.3912
MBP (mmHg)	109.5 (±11.23)	103.8 (±10.83)	109.7 (±6.29)	0.0861
Differential pressure (mmHg)	60.09 (±16.28)	50.14 (±13.62)	52.95 (±15.41)	0.0924

Abbreviations: SD = standard deviation, PI = plaque index, BOP = bleeding on probing, PD = probing depth, CAL = clinical attachment level, HbA1C = glycated hemoglobin, PGT = pyruvic glutamic transaminase, OGT = oxalacetic glutamic transaminase, BMI = body mass index, SBP = systolic blood pressure, DBP = diastolic blood pressure, MBP = mean blood pressure. Bold font = *p* < 0.05, a, b, c = different letter means statistically significant difference between groups (*p* < 0.05). Categorical (C-reactive protein) intergroup comparison was performed using the chi-square test. Other parameters were analyzed using the ANOVA test of repeated measures with Tukey’s post-tests.

**Table 3:** Linear regression analysis of subjects to assess the effects of different time periods on clinical periodontal parameters, biochemical and physical examinations, and blood pressure parameters.

Periodontal Clinical Parameters												
	PI		BOP		PD ≤ 4 mm		PD ≥ 5 mm		CAL ≤ 3 mm		CAL ≥ 6 mm	
	β (95% CI)	*p*-value	β (95% CI)	*p*-value	β (95% CI)	*p*-value	β (95% CI)	*p*-value	β (95% CI)	*p*-value	β (95% CI)	*p*-value
90 days	−0.007 (−0.01–0.001)	0.021	−1.80 (−2.46–1.13)	0.0001	−0.05 (0.02–0.08)	0.001	−0.05 (−0.08–0.03)	0.0001	−0.06 (−0.09–0.03)	0.0001	−0.003 (−0.01–0.005)	0.41
180 days	−0.008 (−0.01–-0.001)	0.021	−1.81 (−2.47–1.14)	0.0001	−0.05 (0.03–0.08)	0.001	−0.05 (−0.07–0.03)	0.0001	−0.06 (−0.09–0.03)	0.0001	−0.003 (−0.02–0.005)	0.40
Biochemical exams												
	HbA 1_c_		Fasting glucose		Total Cholesterol		C reactive protein		PGT		OCT	
90 days	−0.05 (−0.36–0.24)	0.73	−0.004 (−0.006–0.02)	0.39	−0.001 (−0.006–0.003)	0.58	−0.38 (−0.82–0.05)	0.08	−0.005 (−0.03–0.01)	0.56	−0.0007 (−0.2–0.21)	0.94
180 days	−0.05 (−0.36–0.25)	0.73	−0.005 (−0.007–0.02)	0.40	−0.002 (−0.007–0.004)	0.58	−0.39 (−0.82–0.05)	0.08	−0.005 (−0.03–0.01)	0.56	−0.0007 (−0.19–0.20)	0.95
Physical Exams and Blood Pressure Parameters												
	Hip/Waist ratio		MIB		SBP		DBP		MBP		Differential pressure	
90 days	−0.96 (−3.01–1.06)	0.34	−0.001 (−0.03–0.03)	0.93	−0.002 (−0.12–0.009)	0.79	−0.008 (−0.003–0.20)	0.15	−0.004 (−0.009–0.018)	0.53	−0.007 (−0.02–0.003)	0.14
180 days	−0.97 (−3.01–1.06)	0.34	−0.001 (−0.03–0.04)	0.93	−0.001 (−0.12–0.009)	0.74	−0.008 (−0.003–0.20)	0.15	−0.004 (−0.009–0.018)	0.53	−0.007 (−0.01–0.001)	0.14

Abbreviations: PI = plaque index, BOP = bleeding on probing, PD= probing depth, CAL = clinical attachment level, HbA1C = glycated hemoglobin, PGT = pyruvic glutamic transaminase, OGT = oxalacetic glutamic transaminase, BMI= body mass index, SBP= systolic blood pressure, DBP = diastolic blood pressure, MBP = mean blood pressure. Bold font = *p* < 0.05.

**Table 4:** Logistic regression of participants at different follow-up times, associated with periodontal and blood pressure parameters.

	Time 1		Time 2	
	OR (95% CI)	*p*-Value	OR (95% CI)	*p*-Value
BOP (% of sites)				
<30 %	Ref		Ref	
≥30 %	0.02 (0.002–0.29)	0.001	0.05 (0.009–0.26)	0.000
CAL ≥6 mm (% of sites)				
<30 %	Ref		Ref	
≥30 %	0.54 (0.18–1.68)	0.28	0.61 (0.19–1.94)	0.41
SBP (mmHg)				
<140	Ref		Ref	
≥140	0.48 (0.15–1.61)	0.24	0.83 (0.26–2.65)	0.75
DBP (mmHg)				
<90	Ref		Ref	
≥90	1.17 (0.34–1.02)	0.79	1.97 (0.59–6.63)	0.27

Abbreviations: Time 1 = baseline versus 90 days; Time 2 = baseline versus 180 days, OR = odds ratio, CI = confidence interval, BOP = bleeding on probing, CAL = clinical attachment level, SBP = systolic blood pressure, DBP = diastolic blood pressure, Bold font = *p* < 0.05.

## Data Availability

Data generated in this research project are available by contacting the last author of this paper via email. It is stored electronically as Excel worksheets.
